# A Survey of Using Swarm Intelligence Algorithms in IoT

**DOI:** 10.3390/s20051420

**Published:** 2020-03-05

**Authors:** Weifeng Sun, Min Tang, Lijun Zhang, Zhiqiang Huo, Lei Shu

**Affiliations:** 1 Key Lab Intelligent Control & Optimizat Ind Equip, Dalian University of Technology, Dalian 116024, China; wfsun@dlut.edu.cn (W.S.); 786917136@mail.dlut.edu.cn (M.T.); zhljwork@163.com (L.Z.); 2 College of Engineering, Nanjing Agricultural University, Nanjing 210031, China; 3 University of Lincoln, Lincoln 65102, UK; zhuo@lincoln.ac.uk

**Keywords:** swarm intelligence algorithm, ant colony optimization, particle swarm optimization, artificial bee colony algorithm, wireless sensor network, UAV

## Abstract

With the continuing advancements in technologies (such as machine to machine, wireless telecommunications, artificial intelligence, and big data analysis), the Internet of Things (IoT) aims to connect everything for information sharing and intelligent decision-making. Swarm intelligence (SI) provides the possibility of SI behavior through collaboration in individuals that have limited or no intelligence. Its potential parallelism and distribution characteristics can be used to realize global optimization and solve nonlinear complex problems. This paper reviews representative SI algorithms and summarizes their applications in the IoT. The main focus consists in the analysis of SI-enabled applications to wireless sensor network (WSN) and discussion of related research problems in the WSN. Also, we concluded SI-based applications in other IoT fields, such as SI in UAV-aided wireless network. Finally, possible research prospects and future trends are drawn.

## 1. Introduction

For ease of reading, we abbreviate nouns, as shown in Table 1.

In nature, many creatures—such as fishes, birds, and bees—have group behaviors. The abilities of individual members in a group are limited, but the whole group has a strong vitality. The strong vitality is not only a simple superposition of individual abilities but also an adjustment of individual behaviors through exchanging information, cooperating, and finally reflecting group intelligence.

Swarm intelligence (SI) [1] algorithm is a simulation method to simulate biological group intelligence. The potential parallelism and distributed characteristics of SI algorithms enable the possibility of solving complex nonlinear problems with advanced capabilities in terms of self-adaptability, robustness, and search ability. Up to now, there are many SI-inspired optimization algorithms, such as classical particle swarm optimization (PSO) [2] and ant colony optimization (ACO) [3]. In recent years, there exist many improvements—such as artificial bee colony (ABC) [4], bacterial foraging algorithm (BFO) [5], and butterfly optimization algorithm (BOA) [6]. SI algorithms search the optimized solution based on heuristic information. It can be applied to a wide variety of optimization problems (e.g., dynamic optimization problems, multi-objective optimization problems) and NP problems. With the ever-increasing development of IoT, SI exhibits great applicational prospect in IoT-related applications.

**Figure 1:**
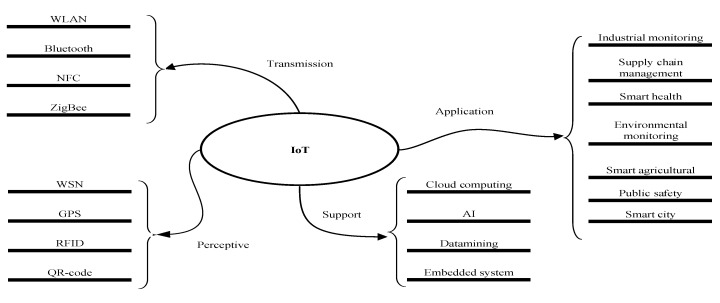
Essential IoT technologies.

IoT is a powerful Internet based on advanced technologies, such as radio frequency identification devices (RFID), sensor technology, cloud computing, and wireless sensor network (WSN) [7]. It allows users to connect any physical object to the Internet for information exchange, communications, and decision-making. However, the development of IoT still faces some challenges. For example, plenty of smart devices from heterogeneous sources can be connected, so security is one of the key challenges in IoT. Also, the vast number of devices in the IoT will cause energy consumption problems and ‘spam’ problems. Furthermore, the IoT system is complex and dynamic; thus, many SI algorithms are developed to compute such complex activities with simple individuals. By modeling the IoT system as a group of simple devices, the SI-based algorithm can be adopted to achieve global optimization. Besides, cloud computing plays an important role in data analysis based on the huge amount of data acquired from IoT systems. SI also applies to the applications of cloud computing, facilitating solving multi-objective optimization problems. In general, the category of IoT can be classified into four parts according to its hot topics and the latest research directions, as shown in Figure 1. 

WSN is an advanced technology in the IoT, which consists of a large number of nodes that have capabilities of sensing, computing, processing, and communicating [8]. Nevertheless, for each sensor, its battery capacity and sensing ability are limited; thus, there are still some crucial problems in WSN, such as routing protocol planning, node localization, and cluster head selection. The SI algorithm emerges from the foraging behavior of the insect community and attempts to find the optimal path of insects. These algorithms have adaptability, robustness, distribution, and extensibility, which is compatible with WSN’s routing protocol requirements. Therefore, many SI-based algorithms have been developed for WSN routing protocols. Among these, the cluster routing protocol is a commonly used WSN routing protocol, in which the cluster head (CH) selection is one of the key problems. Specifically, the CH selection problem is an NP-hard problem. As SI is suitable to solve NP problems, it is applicable to be implemented with few parameters. Also, its multi-objective optimization ability can find CH in multiple clusters at the same time. GPS is often used to solve the positioning problem, but it is not suitable for WSN because of its high energy consumption and unavailability in some specific areas. Node localization can be regarded as an error optimization problem, which belongs to a complex optimization problem, and the SI algorithm can be used to solve this kind of problems.

UAV plays an extremely important role in the field of IoT due to its features of dynamic deployment, convenient configuration, and high autonomy. In general, there are three typical use cases in UAV-assisted communications: (1) UAV-aided coverage, carrying the flight base station, completing the heterogeneous 5G system, and improving the coverage and capacity of wireless access technology; (2) UAV-aided relay, deploying UAVs to provide wireless connections between two or more remote users or user groups without reliable direct communication links; (3) UAV-aided information dissemination and data collection, such as data collection in smart city. The challenges of UAV network lie in the fact that the link is required to be established indirectly due to the variable topology structure, dynamic routing protocol planning, and energy-saving requirements. There exist many UAV applications where SI is used to improve their applicability. For instance, the PSO algorithm is applied to path planning and UAV positioning technology. The design of an individual collaboration of the SI algorithm is compatible with UAV, so the UAV swarm was later proposed.

Many studies concentrate on the summarization of the principles and applications of SI algorithms in the WSN. In 2011, Muhammad Saleem et al. [9] Reviewed SI-based routing protocols in WSNs. In their study, they proposed a WSN routing protocol framework based on SI and classified WSN routing protocols, which include multiple/single routing, reactive/active/hybrid routing, source/next-hop routing, plane/layered routing, data/address for the center routing, distributed/centralized routing, best-effort/QoS-aware routing, the event-driven/query-based routing, using energy-aware or not, packet path if there is a cycle, fault tolerance, and load balancing, and compared WSN routing protocols based on SI and non-SI. In 2015, Sandra Sendra et al. [10] studied systems based on animal and natural behavior in WSNs; their articles classified systems according to animal species and analyzed the usage trend of each type of system. In 2018, Zedadra O et al. [11] reviewed various SI algorithms according to the inspiration source and algorithm maturity. Also, their potential application scenarios were also summarized.

Although some studies summarized relevant improvements in SI-based applications, most of them only consider ant colony and bee colony algorithms. Some algorithms proposed algorithms, such as PSO-based routing algorithms, are ignored. The scope of Muhammad’s survey mainly focused on routing protocol in WSNs; however, it does not discuss sensor deployment and CH selection problems. In Sandra’s study, they considered a variety of bioinspired systems in WSNs [10], but there were few comparative analyses of different approaches to the same problem. In Zedadra O’s study, they presented an elaborate introduction to the classification of SI [11], but it lacks the analysis and comparison of related methods towards similar problems.

To solve these problems, this paper summarizes the application of SI algorithm in the IoT. Our paper generally classifies the network types into three categories: (1) the application of SI in the WSN which is then divided into five sub-problems in terms of CH selection, routing protocol, UAV application, sensor deployment, and node location; (2) the application of SI in UAV-aided wireless network; (3) the application of SI in other related IoTs. According to the role of UAV in the network, UAV-aided wireless network is classified into three categories: UAV as a base station (DBS), UAV as relay and UAV assisted information collection, respectively. Besides, we presented some widely applied SI algorithms, such as PSO, ACO, and ABC.

The main contributions of the survey are outlined below:

- This paper introduces and summarizes more than 40 applications of SI in IoT—such as the WSN and UAV network, especially in the last three years;
- This paper compares 11 SI-based CH selection algorithms and 13 SI-based routing protocols in WSN applications;
- We summarize the validation platform and parameter selection of each method used in WSNs as well as their merits and demerits;
- We survey the state-of-art SI algorithms in IoT applications and discuss potential future development directions.

The rest of this paper is organized as follows: Section 2 introduces the principle of SI algorithm. Section 3 analyzes and compares different applications of swarm intelligence algorithms in WSNs. Section 4 presents the application of SI in UAV-aided wireless network. Section 5 summarizes the application of swarm intelligence algorithm in other Internet of Things. Section 6 concludes the verification scenarios and verification platforms adopted. Section 7 discusses the current state of the SI algorithms in the wireless network and forecasts their future development. The conclusion is drawn in Section 8.

## 2. Swarm Intelligence Algorithms

**Figure 2:**
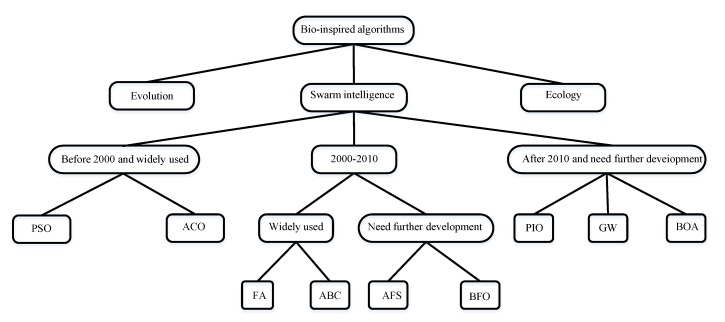
Taxonomy of SI-based computation algorithms.

The SI algorithms were designed to study the principles of simple individuals produce that can exhibit advanced and complex swarm optimization behaviors through cooperation, organization, information exchange, and learning among individuals in a swarm [12]. In Figure 2, the SI algorithms are classified according to their historical development and related applications. To be more specific, the SI algorithms were initially proposed in the 1990s, and now their applications have been largely examined and are relatively mature, including ACO by Dorigo (1992), and PSO by Kennedy and Eberhart (1995). SI algorithm proposed from 2000 to 2010 includes Bacterial Foraging Optimization (BFO) by KM Passino (2002), Artificial fish-swarm Algorithm by XL Li (2002) [13], artificial bee colony (ABC) algorithm by Basturk and Karaboga (2006), firefly algorithm (FA) by Yang (2008) [14]. Newer and promising SI algorithms, including pigeon, inspired optimization (PIO) [15] by Duan (2014) [12], grey wolf optimizer (GW) by S. Mirjalili and A. Lewis (2014) [7], butterfly optimization algorithm (BOA) by S. Arora and S. Singh (2015). In the following, the basic principles of these widely used SI algorithms are briefly introduced.

### 2.1. Particle Swarm Optimization (PSO)

**Figure 3:**
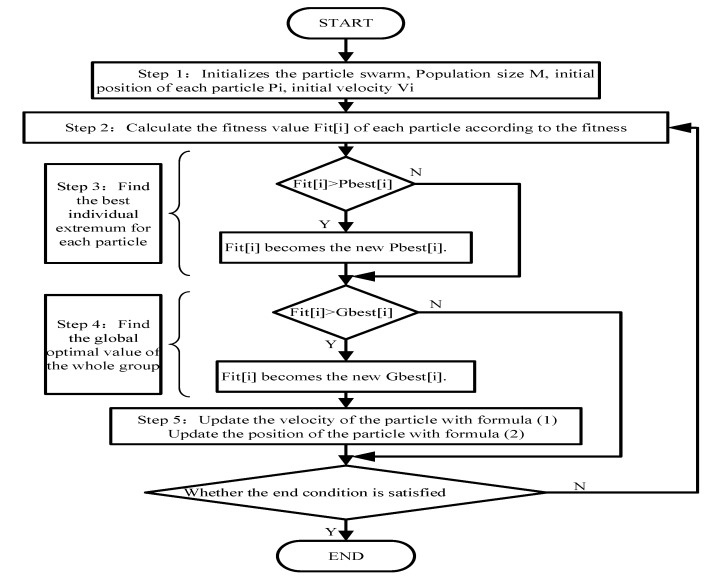
Flowchart of the PSO algorithm.

PSO algorithm is a SI global random search algorithm proposed by Kennedy and Eberhart, which simulates migration and swarm behavior during foraging. Each individual in the flock aggregation model follows the following rules: (**a**) avoiding collisions with neighboring individuals; (**b**) matching individual velocity in the neighborhood; (**c**) flying to the center of the flock, and the flock as a whole flies to the target. In the PSO, the potential solution to each optimization problem is a bird in the search space, called a particle. All particles have a fitness value of the particle itself determined by the optimized function, and each particle has a speed that determines its direction and distances. The particles then follow the current optimal particle through the solution space. Figure 3 is the flow chart of the standard PSO algorithm.

In Figure 3, the Pbest [i] indicates the optimal position found by particle i, called individual extremum. Gbest [i] is the global optimal position found by the whole particle swarm search. In Step 5, particle velocity update Formula (1) and position update Formula (2) are

(1) $$V_{\mathrm{id}}={\omega V}_{\mathrm{id}}+C_{1}\mathrm{random}\left(0,1\right)\left({\mathrm{Pbest}}_{i}-X_{\mathrm{id}}\right)+C_{2}\mathrm{random}\left(0,1\right)\left({\mathrm{Gbest}}_{i}-X_{\mathrm{id}}\right)$$

(2) $$X_{\mathrm{id}}=X_{\mathrm{id}}+V_{\mathrm{id}}$$

Here, ω is an inertia factor, and its value is non-negative. The larger ω is, the stronger the global optimization ability is, and the weaker the local optimization ability is. $C_{1}$ and $C_{2}$ are acceleration constants the former is the individual learning factor of each particle, and the latter is the social learning factor of each particle.

### 2.2. Ant Colony Optimization (ACO)

In ACO, each ant releases pheromones on the path. The whole ant colony can perceive pheromones. The ants in the ant colony will choose the path with higher pheromones, and ants passing through the path will release pheromones so that after a while, the whole ant colony can follow the shortest path to reach the food. The advantages of ACO include strong global optimal ability and flexibility in implementation. It is suitable for combining with other algorithms. The formulas of ACO are as follows, and detailed explanations of parameters are included in Table 2.

Transition probability $P_{ij}^{k}\left(t\right)$ calculation Formula:

(3) $$P_{ij}^{k}\left(t\right)=\{\begin{array}{c} \frac{{\left[\tau_{ij}\left(t\right)\right]}^{\alpha }\cdot {\left[\eta_{ij}\left(t\right)\right]}^{\beta }}{\sum_{s\in J_{k}\left(i\right)}{\left[\tau_{is}\left(t\right)\right]}^{\alpha }\cdot {\left[\eta_{is}\left(t\right)\right]}^{\beta }}\;,if\;j\in J_{k}\left(i\right)\\ 0\;,otherwise \end{array}$$

Heuristic factor calculation Formula:

(4) $$\eta_{ij}=\frac{1}{d_{ij}}$$

Pheromone calculation Formula:

(5) $$\tau_{ij}\left(t+n\right)=\left(1-\rho \right)\cdot \tau_{ij}\left(t\right)+\Delta \tau_{ij}$$

(6) $$\Delta \tau_{ij}=\overset{m}{\underset{k=1}{\sum }}\Delta \tau_{ij}^{k}$$

(7) $$\Delta \tau_{ij}^{k}=\{\begin{array}{c} \frac{Q}{L_{k}},\;if\;ant\;k\;passes\;the\;edge\;\left(i,\;j\right)\;during\;this\;trip\;\\ 0,\;otherwise \end{array}$$

### 2.3. Artificial Bee Colony (ABC)

**Figure 4:**
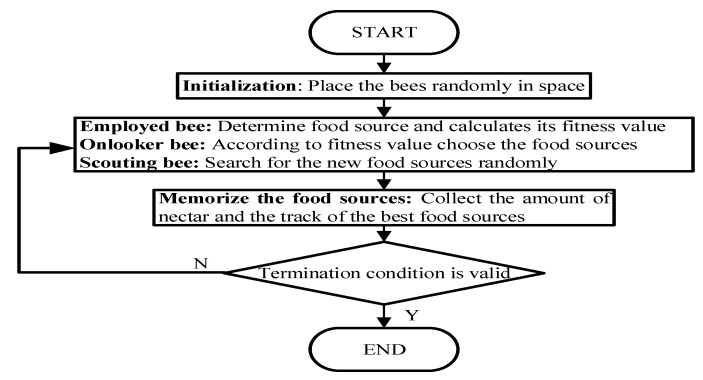
Flowchart of the ABC algorithm.

The ABC simulates the honey gathering behavior of bee swarm, and bees have different behaviors according to their respective division of labor and realize the information communication and sharing of bee swarm to achieve the optimal solution. The artificial bee colony algorithm divides the artificial bee swarm into three categories: employed bees, onlookers, and scouts. In each search process, guiding bee and following bee mine food source successively, that is, search for the optimal solution. While scouting bee observes whether it is trapped in local optimum, and randomly searches for other possible food sources if it is trapped in a local optimum. Each food source represents a possible solution to the problem, and the amount of nectar from the food source corresponds to the quality of the solution. Figure 4 is the flow chart of the ABC algorithm.

The difference between artificial bee colony algorithm and other SI algorithms consists in the role of bees that can be reversed. That is, if the food source information does not improve after several iterations, the food source information should be stopped, and thus the bees should become scouting bees. The advantages of ABC lie in strong global search ability and fast convergence speed. Its disadvantages includes the diversity of race is poor. When the solution is close to the global optimum, it is easy to fall into the local optimum, resulting in a stagnation phenomenon [16,17].

Besides, some other SI algorithms are useful, such as bacterial foraging optimization (BFO) which simulates the behavior of bacterial populations, including three steps: chemotaxis, reproduction, and dispersal, butterfly optimization algorithm (BOA) which simulates the butterfly population to find food. Each butterfly emits a certain concentration of fragrance. Each butterfly senses the smell of other butterflies around it and moves towards the butterfly that emits more fragrance.

## 3. Applications of Swarm Intelligence Algorithms in WSN

**Figure 5:**
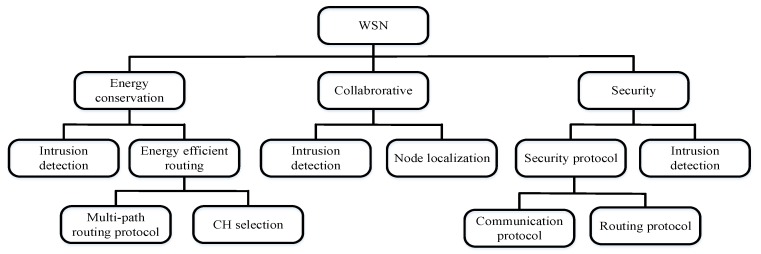
Main problems in WSNs.

WSN is the foundation of the future network IoT. It is a wireless network composed of a large number of static or mobile sensors in a self-organizing and multi-hop manner. WSN can collect, process and transmit the detected information of the objects within the coverage of the network and report to users. Due to its characteristics of low cost and high adaptability, it has attracted extensive attention in recent years and has been widely applied in varied fields, such as military, agricultural production, ecological environment monitoring, industry, and medicine. At present, the main goal of WSN design is to improve performance, save energy and ensure secure communication. Figure 5 shows the key problems in WSNs. According to the type of problems in WSNs, this section is divided into SI-based CH selection algorithm, SI-based WSN routing protocol, and SI-based WSN other applications.

### 3.1. CH Selection Algorithms Based on SI

Routing protocols in WSNs can be divided into planar routing protocols and hierarchical routing protocols according to communication logic [18]. In hierarchical routing protocols, networks are split into clusters of different sizes. Each cluster consists of a CH and several members of the cluster. The cluster head manages or controls the member nodes in the whole cluster, coordinates the work among member nodes, takes charge of the collection of information in the cluster, the fusion processing of data and the forwarding between clusters. Sensor nodes send their perception data to the associated CH. Then, CHs add them up and send them to a BS or the sink node. Therefore, the CH energy consumption is generally faster.

The CH selection is a key problem in WSN design. In this paper, the CH selection method is classified according to sensor coverage mode, clustering method, whether the number of clusters has changed, and the mobility of CH, whether the size of clusters has changed, Intra-cluster communication method and factors for selecting CH. The explanation of these classification methods can be found in Section 3.1.1. Also, they are compared with low energy adaptive clustering hierarchy (LEACH), a classical hierarchical routing protocol that does not employ SI methods. The basic idea of LEACH is to randomly select cluster head nodes in a cyclic manner and distribute the energy load of the entire network to each sensor node evenly. Network energy consumption will decrease, and the overall network survival time will increase. The explanation of classification attributes is described below:

#### 3.1.1. Cluster Classification Properties

- Sensor coverage mode

Divide sensor node coverage into two types: deterministic coverage and random uncertainty coverage. Random uncertainty coverage refers to the random distribution of sensor nodes. The distribution of sensors controls deterministic coverage.

- Clustering method

Classify the clustering methods into static clustering and dynamic clustering. In the static clustering network, the network is divided into many clusters. In dynamic clustering, a cluster is passively created near the CH node.

- The number of clusters

The number of clusters represents the number of clusters per turn. There are two types: fixed (F) and variable (V).

- The size of the cluster

The size of the cluster refers to the maximum path length from the CH to other nodes in the cluster. There are two types: fixed (F) and variable (V).

- Intra-cluster communication method

Inter-cluster communication means communication between sensor nodes and communication between sensors and CH. There are two types: multi-hop jumps and single-hop, depending on whether multi-hop communication is used.

- CH selection factors

The main factors that need to be considered when selecting CH: residual energy (E), distance to sink (D2S), distance to BS (D2B), cluster center (C), distance to other nodes (D2N).

#### 3.1.2. CH Selection Algorithms Based on PSO

Literature [19] proposed a hybrid algorithm of PSO and Tabu named Tabu-PSO to select the CH with the lowest energy consumption in the cluster and to improve the ability to select CH in the WSN. Clusters are formed by the distance from the node to the BS and the energy level of the node. Tabu search was used to improve the racial diversity of PSO to avoid local minimum problems by increasing the number of clusters and improving the survival rate of nodes. Compared with LEACH and PSO, their proposed algorithm can effectively reduce the average packet loss rate and average end-to-end delay. 

Reference [20] proposed a PSO based WSN routing algorithm named EPMS, which combines virtual cluster and mobile reception technology. Compared with Tabu-PSO, this algorithm considers not only residual energy but also node position (distance from a node to the center of gravity) for selecting CH. After the mobile sink visits the entire cluster heads, selecting the node whose residual energy is larger than the average residual energy of all nodes in the cluster as a new CH. The results demonstrated the improvement in prolonging the network life and reducing the average delivery delay.

Reference [21] proposed a PSO-based CH selection algorithm and a cluster formation based on weight function. Different from the general cluster formation method that only considers the distance from non-CH to CH, this method uses the weight function (e.g., parameters include the distance to CH, the residual energy of CH, the distance from CH to BS) to form the cluster. Although the algorithm takes into account the WSN capacity balance and fault tolerance, it only applies to isomorphic networks.

In [22], a method based on fuzzy clustering and PSO was proposed to reduce the number of sensors that cannot connect to the CH and the number of CHs that cannot connect to the BS in 3D-WSN. In their method, a function of the total number of unconnected sensors in all clusters is designed as the objective function of the PSO algorithm to find CH, and then the optimal CH is used in the improved FCM (fuzzy c-means) algorithm. 

Reference [23] proposed an algorithm using BFO to overcome the premature convergence of PSO. The algorithm minimizes the distance between CH and cluster members compared with the LEACH algorithm, their proposed algorithm can improve the network life by reducing the total energy consumption.

The literature [24] proposed a clustering method using PSO (EC-PSO) in a square heterogeneous network. It is to avoid the generation of energy hole and to select CHs by searching the energy center. Their method assumes that the nodes near CH also require a lot of energy, so CH should be located in the energy center. The algorithm adopts a mobile data collector to avoid hot spot problem, but it may result in packet losses. Also, the method has good performance in energy consumption and network life, but the data transfer needs to be improved.

#### 3.1.3. Cluster Head Selection Algorithm Based on ACO and ABC

Reference [25] proposed an improved ACO algorithm to optimal mobile sink movement track. The movement of mobile sink between CHs is regarded as a TSP problem, and the improved ACO is used to find the optimal path to access all CHs through a single-hop physically close communications. To save energy, rotate CH only when any CH residual energy falls below the energy threshold. This method improves the network lifetime and data delivery, but only grid partition is used for cluster partition, without considering the uniform distribution of cluster nodes.

Reference [26] proposed a hybrid MAC, routing and unequal clustering cross-layer protocol (FAMACROW) based on fuzzy and ant colony algorithm. The model used the Mamdani fuzzy reasoning system (FIS) to select CH. Input parameters: residual energy, neighborhood proximity, and link quality indicator. The reliability of the protocol has improved with the use of link quality indicator (LQI) to select CH and transfer the probability of route between clusters. Simulation results showed that the energy consumption of CH is reduced and the node survival time is extended.

Reference [27] proposed a multi-path routing in the WSN by using the Fractional ABC (FABC) and the exponential ACO (EACO). The first step is to use the FABC to find the CH by considering some parameters, such as energy, distance, and delay. In the second stage, the proposed new algorithm EACO (index ACO) is adopted to realize the multi-path path discovery. Compared with other SI hybrid algorithms, the EACO-FABC has better performance in energy and goodput. Also, it can easily adapt to the IEEE 802.15.4 protocol (a low-rate communication protocol). However, as can be seen from the simulation diagram, the CH distribution is very uneven.

Reference [28] proposed a centralized cluster routing protocol based on Sugeno fuzzy reasoning system named LEACH-SF. The ABC is used to adjust fuzzy rules. Each optimization is performed before LEACH -SF runs. The results showed that the LEACH-SF is superior to the existing fuzzy-based clustering algorithms in terms of minimizing the distance within the cluster, maximizing the network lifetime and maximizing the number of packets received by the BS. The LEACH -SF also has the potential to be expanded in mobile sensor nodes and multi-hop routing. The simulation is based on a heterogeneous network.

In [29], the authors proposed an improved ABC named iABC. Therefore, iABC used a Student’s *t* distribution to improve the population’s initialization and used differential evolution to improve the solution search equation. The method enhances global convergence by using a compact student T distribution. Simulation results showed that this method consumes less energy and delivers the most packets with minimum end-to-end delay. When the BS is placed outside the network field, the impact on the performance of the protocol is also small.

Table 3 compares the CH selection algorithms.

With respect to CH selection, clusters can be formed in two ways: (1) First, the family of clusters is divided, and then the CH is found; (2) For the former, the initial CH is usually selected as the node at the center of the cluster. While the latter at the CH when the choice to consider to whether the CH uniform distribution, the size of the cluster (high node can lead to CH burden too big, reduce energy consumption too fast, accelerate CH death) for the CH rotation, now more literature or rotation as the standard with CH energy is lower than the threshold value also has to rotate according to time, but we think the former is more flexible, now for the development direction of intelligent sensors (which can switch of receiving to save energy) or consider QoS of WSN has more practical value. In addition, the WSN is divided into single-hop and multi-hop within the cluster. The multi-hop WSN within the cluster should also consider those member nodes that are close to CH and will consume energy faster. In this case, one possible solution is to use the mobile sink to seek the energy balance. According to [25], CH transformation is based on "significant differences in energy in different regions", which can enable preventing relay nodes from dying too quickly. 

In the SI-based CH selection algorithm, it heavily relies on the performance of selected SI algorithm to locate the optimal solution and the use of SI to find the CH according to the requirements (parameters such as residual energy and distance of nodes). It is important to combine SI algorithms with other approaches to improve the analytic performance further. For instance, the PSO algorithm is limited to giving the local optimum solution; thus, using other algorithms can prevent the PSO from falling into local optimum.

### 3.2. WSN Routing Protocols Based on SI

Different from traditional wireless networks, the scale of WSN is often very large, and nodes are often randomly deployed in the network. The WSN still has limited capabilities in a node or a network. For example, each node has limited calculation and communication capabilities, and its energy is often restricted. Therefore, the efficient design of routing protocols needs to take into account the communication path between sensor nodes (source) and the command center (receiver) to save energy. Furthermore, protocols must be self-organizing and robust to failures and losses. The SI algorithms have promising characteristics—such as self-adaptability, robustness, and search ability—to solve complex non-linear problems, which are key attributes required by the WSN. Therefore, a growing number of SI-based routing algorithms have been developing in WSN routing. In this paper routing protocols according to distributed (D)/centralized (C), Besteffort (B)/QoS (Q), single path (S)/multipath (M), event-driven/query-based routing, routing fault tolerance, energy awareness, loop free.

#### 3.2.1. Routing Protocol Classification Properties

- Distributed (D)/Centralized (C)

If the protocol is centralized, the discovery and maintenance of routing information are controlled by a single node called receiver or base station. In a distributed way, each node collects or builds its own routing information.

- Besteffort (B)/QoS (Q)

Agreements that do not provide any QoS assurance for delivery to the application are classified as the best effort. Protocols that provide quality assurance for application routing services are labeled QoS-aware (e.g., end-to-end latency, latency jitter, available bandwidth, packet loss, etc).

- Single path (S)/Multipath (M) routing

Routing protocols may maintain single or multiple routes to a given destination. Single path routing can find one or more routes and select the best path for data transmission as well as discard the other paths. Multipath routing is a protocol that finds, maintains, and uses multiple paths to transmit perceptual data.

- Event-driven/Query-based routing

The application program based on routing protocol service is divided into event-driven and query-driven. In the event-driven protocol, data routing is initiated after the sensor detects the event. In the query-based protocol, the sink node broadcasts the corresponding query interest, and later the sensor satisfying the query starts the data routing.

- Fault tolerant

The protocol is robust to topology changes and packet loss

- Energy aware

Whether the protocol prioritizes routing based on energy metrics (for example, the remaining energy of nodes on the route).

- Loop free

If the path used by the packet is guaranteed to have no loops, the protocol is called no loops. The protocol must contain explicit mechanisms to check for and avoid possible cycles.

#### 3.2.2. Wireless Sensor Routing Protocols Based on PSO

In [19], a WSN routing optimization algorithm based on PSO and TS was proposed, through optimizing the number of clusters formed and the number of nodes that should exist in a single cluster to prolong the network lifetime and energy efficiency with energy-saving and responsive manners. The routing optimization algorithm can effectively reduce the average packet loss rate and average end-to-end delay, by increasing the number of clusters and improving the survival rate of nodes.

Reference [20] proposed a PSO-based WSN routing algorithm named EMSP, which mainly combined virtual clustering and mobile sink technology in the routing process. The EPMS defines three packet formats: hello packet is used to determine which cluster send data to mobile sink; messages-c is the message directly sent by CH to mobile sink; message-h packet is the message sent by member node to CH. The sink node begins to collect information from the cluster with the highest residual energy. Only when the sink determines the CH, will the member node transmit data to CH. In this case, the member node is very likely to die before the data is transmitted.

Reference [24] improved the routing mode for energy conservation and energy balance because excessive forwarding near CH can result in the energy hole and unbalanced energy consumption, as well as premature death of nodes. In such a case, a low energy protection mechanism is adopted to avoid the weak node becoming a relay node, and a mobile data collector was introduced to collect data. The PSO is used to search the energy center of the network, and nodes that are nearest to the energy center are selected as CHs. The uncertainty of the moving data collector however may cause data loss.

Reference [30] proposed a new method to extend the network life cycle for cluster-based WSN. An optimization method was applied for selecting target nodes of interest. The protocol extends the network life cycle by selecting the target node optimization method and the relay node to alleviate the work of CH. In addition, an improved PSO algorithm is proposed to minimize transmission distance by updating cluster nodes and to optimize network energy consumption. The protocol distributes nodes evenly across multiple clusters.

Reference [31] put forward a kind of bionic PSO fault-tolerant routing algorithm, by extending the existing multi-objective optimization problem optimization method, fast recovery from the path to failure. Based on the determined objective function, the effective value of the objective function calculated by each selected sensor node is optimized to construct k-disjoint multi-path. A multi-path routing algorithm is used to balance fault tolerance and communication overhead. Compared with the canonical PSO, overall energy consumption and average delay are optimized. However, there is a slight disadvantage at the beginning of the iteration because the method needs a large amount of time to stabilize the target function.

#### 3.2.3. WSN Routing Protocols Based on ACO

For the WSN, optimal routing algorithms and routing protocol are the keys to save communication energy and thus prolong the network lifetime. Reference [25] presented a routing algorithm that combines the clustering algorithm, the ACO and mobile sink. The algorithm improves the ACO by optimizing the heuristic factor (visibility)$\;\eta_{ij}$, and uses improved ACO to find an optimal sink movement track, communicate with all CHs, and collect data through a single hop close communication. Compared with other ACO-based standard algorithms, this method can extend the network life. It is worth noting that the more nodes there are, the smaller the advantage is.

Reference [26] proposed a cross-layer protocol (FAMACROW). This protocol combines the idea of energy-saving hierarchical cluster routing and media access and improve its energy efficiency and network life. In this algorithm, the ACO technology was used to carry out multi-hop routing between clusters from CH to MS, and the fuzzy logic and ACO is used to select CH and route paths between clusters. The reliability of protocols is improved by using link quality indicators to select CH and transfer the probability of routes between clusters. FAMACROW is better than the other unequal clustering routing in terms of throughput, goodput, latency, and the network life.

Reference [27] proposed a multi-path routing in WSNs by using the FABC algorithm and the EACO algorithm. The first step is to use the FABC algorithm with the fitness function to find the CH. In the second stage, the proposed new algorithm EACO is adopted to realize multi-path path discovery. The EACO uses an exponential weighted moving average mode control convergence speed of ACO. Compared with other SI+SI algorithms, as the number of rounds increases, FABC+EACO is better in terms of energy consumption and goodput, but the advantage decreases with the increase of nodes.

Reference [32] proposed an improved energy-saving routing algorithm based on ACO, considering location information and search direction. The algorithm considers not only the distance from the node to the next node but also the distance from the node to sink. It also specifies the search range and direction (pointing to sink) for the next node. Simulation results showed that this algorithm is significantly better than other algorithms when the distance between the sink and the target region is far away, and the average energy consumption of this algorithm increases slowly with the increase of simulation times.

Reference [33] proposed a secure routing protocol based on multi-objective ACO (named SRPMA). The goal of this algorithm is to find better multi-path routing results. The main idea of this algorithm is to introduce Pareto multi-objective optimization strategy into the ACO, and to use the improved ACO to take the residual energy of nodes and the trust value of routing path as the routing optimization targets. Compared with related ACO-based security routing protocols, the SRPMA can achieve better performance in terms of packet loss rate, routing load and average energy consumption under black hole attack. Also, the advantage becomes more evident with the increase of the number of black hole nodes because the SRPMA requires additional routing control packets to maintain the trust model and update the trust values of the nodes. 

Large-scale WSNs may contain a large amount of sensor nodes distributed into the area of interest [34]. Reference [35] proposed a routing protocol, named CB-RACO in large-scale WSNs, where the ACO and LP are combined. The goal is to keep data transmission at a high level while minimizing energy consumption. Although this protocol divides clusters but is not CH-based, in order to prevent energy consumption too fast, each ant can only live in its own community (cluster activity). Besides, considering large-scale WSNs, the inter-community retransmission and intra-community multi-path routing strategies are respectively used to improve the reliability and load balance of data transmission. The advantage of this strategy lies in the ability to organize and to run the network autonomously when the network is overloaded whilst the memory cost is very low. The CB-RACO method can achieve high data delivery rates in large-scale WSNs. 

Table 4 compares the routing algorithms. With respect to WSN routing protocol, the following factors need to be considered: first, because the energy of nodes is limited, the routing protocol design should be simple, efficient, and energy-saving and take into account the energy balance to extend the network life cycle. Second, the network can be extended to respond quickly to changes when nodes die, or new ones join. Third, convergence should be rapid. To sum up, from the literature, most routing algorithms in WSN are distributed, and only a few of them support QoS. Besides, the number of energy-aware protocols is relatively large because WSN requires energy saving. Finally, the PSO or ACO algorithms are popular for routing optimization in WSN applications and for multipath communication. The ACO is good at finding the optimal path. The design of WSN routing protocols has to not only take into account security, latency, packet loss rate, fault tolerance, and other issues but also to consider energy consumption, as do routing protocols in general networks. Factors to consider for WSN routing protocols and their solutions: energy saving (increase the number of clusters, prevent energy cavity made relay protection mechanisms to prevent low energy nodes, balance transmission distance, use mobile sinks, optimize multipath routing), average end-to-end delay, packet loss rate, fault tolerance (multipath routing), and safety.

### 3.3. SI Applications in Sensor Deployment

Reference [22] proposed a method based on fuzzy clustering and PSO to reduce network interruption. Compared with the traditional FCM method, GA and PSO algorithms were introduced to optimize FCM. The hybrid FCM-PSO algorithm is repeatedly executed until the optimal topology of the sensor is determined. Simulation results showed that this method can reduce energy consumption and improve the connection rate of CH to BS, non-CH to CH.

Literature [36] proposed a PSO-based coverage algorithm in static WSNs with randomly deployed sensors. This method partitions networks into grids and calculates the coverage rate of each grid. Constructing a wireless sensor network to fully cover critical grids by deploying minimum sensors on grid points is NP-complete [37]. Then, it adjusts the node sensing range until the coverage rate is higher than 90% and also adjusts the sensing radius with PSO. Compared with traditional PSO-based coverage algorithm methods, this method improves coverage and reduces energy consumption. However, this method may not be suitable for networks where obstacles exist.

In [38], an ACO deployment problem-solving method combined with local search (LS) was proposed. This approach considers the deployment of a WSN that meets the specified minimum reliability level within the task time at the minimum network deployment cost. In their paper, it proposes the problem of a minimum cost reliability constraint for node deployment, proves that the problem is NP-complete, and proposes a local search algorithm. It proved that the ACO solution exhibits better quality than the greedy algorithm.

### 3.4. SI Applications in Node Localization 

In WSN, location information can be used for target tracking, network topology construction. Positioning technology is the basic technology of WSNs, which plays a critical role in supporting the follow-up work and application of WSNs [39]. 

Reference [40] proposed dimension-based PSO (DPSO) and hybrid DPSO (HDPSO) for node positioning in a 3D environment. The DPSO adopts dimension-based optimization technology to determine the location of the target node by considering each dimension, which works well in all applications. HDPSO adopts the grouping technology based on dimension estimation to realize rapid convergence, and it is suitable for the network scene of intensive deployment. The simulation scenario considers noise measurement, signal irregularities, and other interference factors. Compared with conventional PSO-based methods, the DPSO and HDPSO have better positioning accuracy and average time.

Reference [41] proposed a butterfly optimization algorithm (BOA) for node positioning in WSN. The BOA algorithm is used to find the optimal position of the local node. By comparing this method with the PSO algorithm and FA algorithm in terms of positioning error, positioning node, and calculation time. The results showed that the BOA based node positioning scheme has better calculation time and accuracy.

### 3.5. SI Applications in UAV-Aided WSNs

For WSN, the UAV can assist the communication between sensor nodes and assist the positioning of mobile network nodes. UAVs have been widely applied to the WSNs. To minimize the energy consumption and maximize the network life, it is necessary to determine the optimal flight path of UVAs, maximize the value of collected sensing information, and minimize the total cost of flight time, energy consumption and operational risks of UAV under given environmental conditions.

Reference [42] proposed a multi-objective utility function to obtain the optimal UAV flight path and a dynamic VSI updating mechanism. This method combines the GA and ACO to select the optimal path from the possible flight path according to sense, energy, time, and risk-utility. The combination of GA and ACO algorithm divided the whole sensor field into cell regions, and first considered sensor information acquisition (SIG) units. The value of sensing information collected by this path is maximized, and the total cost of flight time, energy consumption, and operation risk of UAV are minimized under given environmental conditions.

Different from static WSNs, mobile WSNs often have more dynamic topology and lower power consumption ability. The most critical issue in mobile WSNs is the mobile aggregation scheduling problem. Reference [43] proposed a BFO-based cooperative mobile sensing scheme in mobile WSNs using UAV as a mobile sink. The process of *Escherichia coli* moving in BFO was used to simulate the distributed self-organization and adaptive displacement of the mobile sink. The UAV decided to move in a specific direction according to the process of *Escherichia coli* flooding. Simulation results showed that this method can reduce energy consumption, reduce delay, and improve the throughput and coverage of network.

### 3.6. Parameters to Be Considered in the Validation of SI Applications in the WSN

In WSN applications, many indicators are considered for evaluating the system’s performance. Commonly used parameters are summarized in Table 5, such as energy consumption, survival time, and throughput [44,45].

From Table 5, it can be seen that most WSN applications take into account the energy consumption problem. For routing protocols in WSN, there are some crucial parameters, such as energy-saving, delay, fault tolerance, and QoS. However, these kinds of literature generally only consider two or three factors, and none of them has considered all factors comprehensively. In future protocols, readers can refer to these parameters shown in Table 5 to provide an optimal routing protocol by considering comprehensive indicators.

## 4. Application of Swarm Intelligence Algorithms in UAV-Aided Wireless Networks

**Figure 6:**
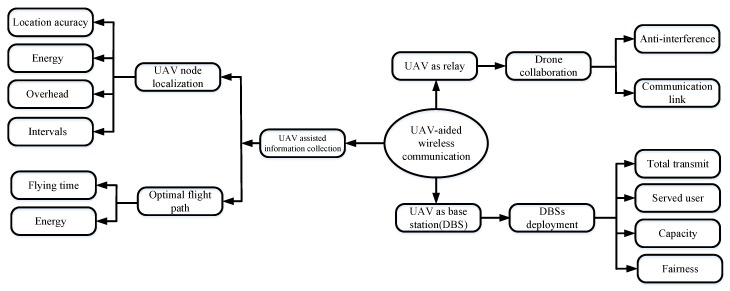
UAV-aided wireless networks.

According to previous literature, the UAV application in the wireless network can be divided into three conditions: (1) in the base station or network congestion problem, the UAV as a temporary base station (DBS); (2) when two or more users distance is far, the UAV can be used as a relay with a wireless connection; (3) as the application of the sensor, the UAV can be used for collecting sensory information. According to these three applications, we further discuss what problems are more inclined to solve in a specific application, and we also analyze some factors that should be considered for each problem, which are illustrated in Figure 6.

For improving the flexibility of the network, the UAV can be integrated into the wireless network as a flight base station (BSs) named DBSs. DBSs are a promising application in the wireless network. However, the deployment of DBSs can face several challenges. First, a 3D-DBSs layout is needed to meet the dynamic requirements of the system. Second, the availability and related resource allocation issues of reliable wireless backhaul links. The final problem is the association between the users and BSs [46].

Literature [47] put forward an algorithm to find an efficient 3D layout of DBSs in addition to the user-BS associations and wireless backhaul bandwidth allocations to maximize the sum logarithmic rate of the users. This is the first time to consider the 3D layout of backhaul resource allocation, user associations, user types, and DBSs simultaneously. User-BS association and wireless back trip bandwidth allocation are found by the decomposition method, and the location of DBSs is updated by the PSO algorithm. 

Reference [48] presented a fair-sense multi-DBS 3D deployment based on PSO, which maximizes the logarithmic sum of user accessibility. The PSO algorithm is adopted in which the stop criterion is selected as the condition for the particle to converge to a certain point. The method achieves optimal fairness with the least number of DBSs deployments. Also, the proposed algorithm can work in networks with different user distributions.

Reference [49] studies the problem of providing wireless coverage when a UAV is used to distribute users on each floor of a high-rise building evenly. PSO algorithm is adopted to find an efficient three-dimensional layout of UAV. When the same number of users is evenly distributed on each floor, the total emission power required to cover all indoor users is minimized. The convergence speed of the algorithms for different building are considered as the evaluation criterion. However, this algorithm is only based on equally distributed users.

Reference [50] proposed an SI-based 3D positioning algorithm (SIL) and SI-based energy saving clustering algorithm (SIC). SIL algorithm follows the boundary-based optimization method and uses the PSO algorithm to detect the location of target UAV nodes. The use of boundary box technology reduces the initial search space and speeds up the convergence rate; bounding box technology was used to refine the position estimation further. The SIC adopts an improved PSO to select CH. The SIL algorithm improves convergence speed and positioning accuracy at a lower computational cost. Comparably, the SIC algorithm is superior to typical routing protocols in packet transfer rate, average end-to-end delay, routing overhead, and energy consumption.

The UAV-assisted wireless network is a direction of future network and IoT. As the DBS and UAV can assist network coverage and act as temporary networks in disaster situations. they can be used as relay, auxiliary information collection, and so on in many kinds of IoTs. The key issues of UAV-aided wireless network are deployment and path optimization. For deployment issues, it consists: (1) joint optimization of deployment and bandwidth allocation to reduce communication delay; (2) deployment of perceived obstacles to improve network coverage; (3) association between users and DBSs. Such issues should be evaluated with regard to user coverage, robustness, and power consumption. With respect to path optimization, energy constraints, obstacles, and perceived information value maximization should be considered, as well as collaboration issues for multiple UAVs. With the development of edge computing, future UAVs should also take into account the data analysis.

## 5. Application of Swarm Intelligence Algorithms in Other IoTs

The VANET is a special form of MANET (mobile ad-hoc network). Multicast routing under QoS constraint is proven to be NP-complete. Therefore, reference [51] transformed the QoS constrained multicast routing problem of VANETs into an SMT (Steiner minimum tree) search problem, and designed a micro-ABC (MABC) [52] method based on binary representation to solve the SMT problem effectively. The method consists of a tiny colony, saving computation time per cycle compared to using a normal swarm size. Simulation results showed that the MABC can find the optimal solution under four destiny nodes, and the increase of destiny nodes has little effect on run time.

Reference [52] proposed an efficient ant colony based routing algorithm for the IoT. The algorithm is more efficient, scalable, and adaptable to network parameter changes, taking energy into account. In this paper, considering the IoT systems as a graph G and turning the IoT routing problem into a TSP-like problem, they use ACO to solve the problem. Simulation results showed that the proposed method can reduce energy consumption, end-to-end delay, and extend node life.

Reference [53] presented a multi-objective PSO (MOPSO) cloud agent scheme. This scheme optimizes resource selection in cloud computing by optimizing the cloud agent (the middle layer between the client and the cloud service provider in cloud computing), using multi-objective PSO to find Pareto optimal solution of cloud agent problem. Compared with GA and a random search algorithm, MOPSO reduces response time, and energy consumption and improves profits. However, as iteration increases, the profit of MOPSO is almost the same as the random search algorithm. 

Reference [54] showed an optimization model for virtual machine selection in cloud IoT health service applications to effectively manage and integrate a large amount of data in industry 4.0. The GA, PSO, and PPSO are both considered for optimization of virtual machine selection. The proposed model can optimize data storage requirements by reducing the stakeholders’ request execution time, turnaround time, and waiting time of medical requests (tasks). The simulation result showed the proposed model demonstrated its superior to the existing model in terms of overall execution time and real-time data retrieval efficiency.

When the IoT devices transmit and exchange data, attackers can easily get detailed information about shared data. Based on the MOPSO [54], literature [55] proposed a security processing method adopting hierarchical clustering and PSO. Its purpose is to find the optimal particle as Pareto solution and hide the secret information innocently. The main idea is that each particle is given a probability, thus increasing the diversity of global optima and preventing it from falling into local optima. Compared with other multi-objective algorithms and GA—based algorithms, the HCMPSO can have the lowest hiding failure in most cases.

Reference [56] proposed a PSO-based non-invasive load scheduling method for the residential user center. It is characterized by edge computing and drives the computing power of the Internet edge and IoT data sources to optimize cloud computing technology. The model is based on the historical power consumption trend, and user satisfaction data in the smart home environment and the constraint PSO is used to load the data. It not only considers the load dispatching of the state grid but also considers the local renewable resources, which is beneficial to the safe development of the power grid.

An important problem in the IoT is to reduce the number of hops from the sensor to the sink. Reference [57] studied the modeling problem of small-world networks with multiple convergence points, and proposed a PSO-based novel shortcut addition algorithm for multi-sink IoT (SAPS). Each cluster is treated as a single aggregate network. The fitness function is composed of the average path length and load to sink. The GA is introduced to update particles through crossover (retaining some information about the current particle and its optimal particle) and mutation (generating new individuals). Simulation results showed that the SAPS can ensure that the average path length of the whole network is short and the load balance between sink nodes is stable.

## 6. Analysis of the Verification Methods Adopted by SI in WSN and Problems

This section presents the verification method in SI-based WSN applications and highlights some key problems. Table 6 concludes the parameters of the simulation platform according to its changes to the scene, such as the change in the number of nodes in WSN, in the area size, and in the location of the base station. The simulated scene types are summarized and compared in Table 7.

These statistics point out: (1) A third of the papers does not address the simulation platform, so it is hard to recreate the experiment; (2) Experimental results are often compared to earlier methods such as LEACH, not compared to the existing outstanding method; (3) Most of the methods for the simulation environment, only consider the result of a case, or there are only one scene change conditions (e.g., only change the number of nodes). There is hard to know if its approach would have an advantageous outcome in other scenarios; (4) In general, most experiments are carried out on the Matlab platform, and castalia3.2 under omnet4.6 is also used as a simulation framework based on wireless body area network and sensor network. 

## 7. Current Situation and Development Trend

### 7.1. Current Statuses

In this section, we conduct a statistical analysis in two aspects. On the one hand, we analyze the number of related literature using PSO, ACO, and ABC in wireless, WSN and UAV in the past three years (2017–2019) and further analyzed the specific applications of the three algorithms in WSNs. On the other hand, we analyze the measures considered by these SI algorithms in optimization.

**Figure 7:**
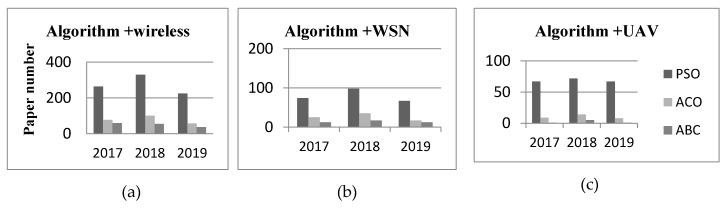
Numbers of SI literatures about wireless, WSN and UAV in Scopus (by October, 2019). The search results by the keywords: (**a**) PSO, ACO, ABC, and wireless; (**b**) PSO, ACO, ABC, and WSN; (**c**) PSO, ACO, ABC, and UAV.

Figure 7 presents related works where SI algorithms (PSO, ACO, and ABC) are applied in wireless network applications in the past three years (2017–2019) (this data comes from Scopus’ search results). 

We can see that the PSO has the largest number of applications in the field of wireless network, even up to two or three times than ACO and ABC literature. The PSO relies on its optimization ability with simple parameters and fast convergence to find the optimal solution to solve the continuity problem, so it is most widely used in function optimization, neural network training, fuzzy system control, and other aspects because of its advantages with fewer and simpler parameters. It is suitable for solving continuous functions. In UAV application, there is a requirement for the positional design between each individual in the flock because the PSO algorithm originally generated to simulate the movement of bird groups. For example, to prevent collisions between individuals, the distance between individuals should not be too close to convenience of communication, individuals should not be too far apart. It is similar to the flight patterns of UAVs, so the PSO can be used in path planning, UAV positioning, task assignment, multi-UAV cooperation. The ACO is originally from ant path-finding, so it is widely used in path planning. The ABC is originally from bee collaboration, so it can be used for multi-UAV cooperation.

**Figure 8:**
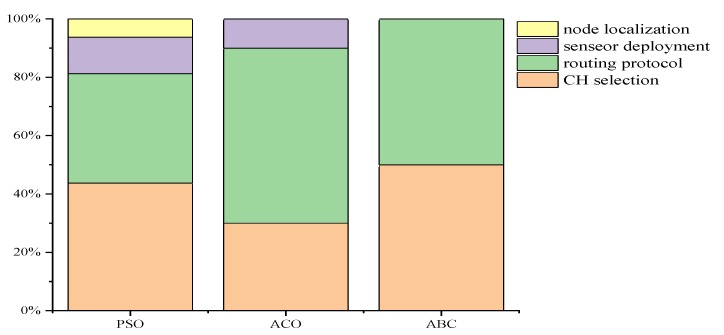
Different applications for PSO/ACO/ABC in WSNs.

As Figure 8 shows, the applications of SI in WSNs mainly lies in CH selection (about 41%) and routing protocol (about 47%). On one hand, many WSN studies focuses on these two problems. On other hand, most SI algorithms can be applied to these two problems. The applications of PSO in WSN is more about CH selection, routing algorithm, and node location. Among them, SI-based node location algorithm adopts PSO more. The ACO has the characteristics of distribution, adaptive optimization, and strong scalability to residual pheromones. Therefore, it is often used to solve routing protocol problems in WSNs. The algorithm has few parameters and high precision. In the optimization, only fitness functions are used as an evolutionary basis. In WSN applications, the ABC has been widely used to solve CH selection, sensor deployment, node location, and other related problems.

**Figure 9:**
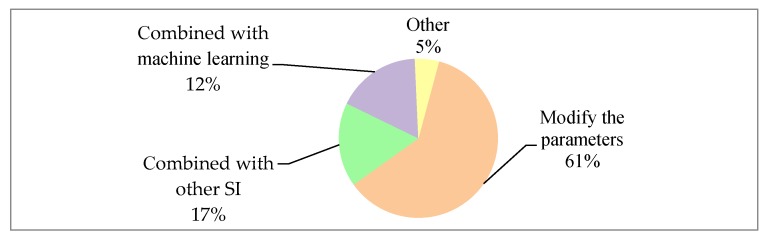
SI algorithm optimization methods.

There are various problems in traditional SI algorithms. Two examples are that the PSO is easy to fall into the local optimal, and the ACO’s early convergence speed is very slow. The optimization of SI algorithms has been considered in the literature we studied. As Figure 9 shows, most kinds of the literatures (61%) currently select improved parameters. For example, the PSO adjusts the inertia factor to adjust the global optimization and local optimization ability. The ACO adjusts the pheromone update factor to improve the convergence rate. Most SI algorithms are easy to implement, so combining them with other algorithms is a good way to improve. For example, many SI algorithms are used in conjunction with other SI (17%), SI is combined with machine learning related technologies (e.g., k-means [58]) and FCM [59]. Other technologies (e.g., LP) can also be combined with SI.

### 7.2. Development Trend

Based on the analysis of state-of-art related works, it believes that the applications of SI in wireless networks has the following development directions: SI has very broad applicational prospects in the WSN. Potential development directions are discussed below.

#### 7.2.1. SI Applications in More WSNs

Reference [27,60] mentioned that the cognitive behavior of artificial bees is completely consistent with the intrinsic dynamic characteristics of cognitive WSN. However, according to the current literature, the applications of SI in a cognitive WSN is relatively rare. The application of ABC in a cognitive wireless network is a direction that researchers can consider.

WSN has become an important part of 5G mobile technology, thereby opening up a new prospect for the advanced application of smart sensors in future IoT applications [61]. Smart sensors are capable of complex processing and analysis of perceived data. In the WSN with smart nodes, if the smart node does not detect an event, it does not need to send a message to CH to save energy, or smart node sensed data for CH events have no effect on test results, it can be suppressed. Therefore, for this kind of WSNs, need to take into account the smart node is the dynamic changes of energy consumption. The energy consumption of different nodes are different, while the commonly used CH selection methods are based on the same energy consumption of nodes in the cluster. In the WSN with smart nodes, CH selection methods have a large development space.

#### 7.2.2. Wireless Network Based on UAV

Due to the wide applicability of UAV, drones have a promising future for assisting wireless networks. In such a network, the problems to be considered are the flight path of UAV, the cooperation between UAV. The characteristic of SI is distributed control, and each individual is directly connected, which is highly consistent with UAV control. At present, the SI algorithm has been widely used in UAV. For example, the PSO is used to locate the optimal solution of UAV, and the ACO is used for optimal path planning of UAV. The next step should be to consider combining the use of drones with actual network scenarios to make drone assistance more targeted.

#### 7.2.3. QoS Assuring on WSN 

The QoS refers to the capability of a network that can provide promising services (including ensuring the transmission bandwidth, reducing the transmission delay, reducing the packet loss rate) for specified network communications using various underlying technologies. Since the traditional OSI model does not apply to wireless networks. Moreover, WSN combines sensor technology and distributed information processing technologies. The traditional QoS system is not applicable to WSNs. More improved QoS system frameworks are needed for WSN. For example, energy conservation and scalability should be emphasized for the physical layer, and the dormancy mechanism should be considered for the sensor to save energy. The network layer should consider the whole network energy consumption balance. In our references, the proportion of literature considering QoS quality is relatively small; thus, how to solve the QoS problem in the WSN is a direction that can be studied.

#### 7.2.4. Intelligent Mobile Edge Computing

As mentioned in [21], the BS in the center has a longer life cycle than in the corner or outside the domain. Moreover, the centralized processing mode represented by cloud computing is unable to handle the number generated by edge devices efficiently. Smart edge proposes that every edge device in the IoT can be equipped with data collection, analysis, computing, communications, and most importantly intelligence. The prominent advantages of the intelligent edge are to reduce latency, reduce bandwidth, reduce duplication, and improve security. However, at present, the intelligent edge also has the problem of resource limitation. Some intelligent edge computing methods can be considered to improve the QoS of wireless networks: (1) real-time use of a UAVs and an efficient means to obtain and analyze data, it is about the UAV task allocation problem, the swarm intelligence algorithm is very good at addressing this issue; (2) only connecting the most valuable data in the edge device to the cloud to reduce data transfer. Ideally, edge nodes send only the absolutely necessary information and make critical decisions as soon as they have access to critical data. For this purpose, appropriate scheduling tasks are needed by considering the priority assignment where SI algorithms are available.

#### 7.2.5. 5G Networks

With the ever-growing development of networks, a 5G network has emerged. Compared with the 4G network, the innovation of the 5G network lies in: micro-base station, beam shaping, and D2D [62]. Reference [58] reported that PSO is used to deal with the problem of joint uplink subcarrier distribution and power control in the D2D underlying cellular network in 5G. Regarding the application of SI to 5G, there may be the following research direction: (1) 5G network is one of the core technology of massive MIMO, due to the intelligent algorithm in solving a large space, global optimum, nonlinear complex problems better than the traditional algorithms, so a lot of application examples of SI in MIMO (especially) based on PSO, follow-up can be further in this direction to massive MIMO; (2) as 5G network is oriented towards a variety of scenarios, different scenarios have different requirements on network mobility, security, delay, and other requirements. Therefore, it is necessary to divide the physical network into multiple virtual networks, which falls into the topic of network slicing technology. However, when a large number of users and devices increase dramatically, the virtual machine resources in the network slice may not meet the demands of users, so the network slice function migration mechanism is strongly required. The migration problem is an NP-hard combinational problem, which is suitable to be solved by the SI algorithm. Moreover, in the 5G-based migration problem, a low time delay has to be seriously considered. Both PSO and ABC algorithms have the advantage of fast convergence speed. We believe that introducing SI algorithms into the 5G migration problem is a feasible and advantageous research direction.

#### 7.2.6. Wireless Security Assured by SI Algorithms 

At present, SI algorithms apply to intrusion detection in network security (e.g., attack source location and cluster analysis), to cryptography (e.g., block cipher), and to reduce computation in a cryptographic algorithm [12]. The characteristics of distributed SI and strong robustness have great development prospects in network security. In the future, it is suggested to improve the global optimum ability and convergence speed of the SI algorithms and reduce the alarming rate of intrusion detection. 

## 8. Conclusions

With continuing advancements in IoT technologies, the number of IoT access devices is rapidly increasing. In this case, 5G networks with low latency, high speed, and high capacity as well as good QoS have become challenges. With the fast-growing 5G, the IoT becomes more complex and heterogeneous but more promising. The WSN is a key technology in the IoT. It is of great importance to support monitoring tasks in large-scale areas. At present, due to the limited energy of WSN nodes, the key problems lie in efficient node selection (CH selection) and node collaborative (e.g., localization problem). The SI algorithms can transform complex NP-problems into individual adaptability to the environment to obtain the optimal solution iteratively. Therefore, it is believed that the SI algorithm enables solving many specific problems in WSN applications. Also, the UAVs have considerations collaboration between individual UAVs that are similar to biological communities. They are crucial mobile and flexible IoT devices that can assist IoT and wireless cellular networks (e.g., 5G) in the future. 

This paper reviews representative SI algorithms and summarizes their applications in the IoT. We consider the applications of WSN and UAV-assisted wireless networks as examples. Also, we discuss the key issues in WSN applications and highlight the potential usability of SI algorithms in optimization problems, such as the CH selection problem. Moreover, the simulation platform and scenarios mentioned in related studies are summarized. We generally divide the UAV-aided wireless network into three categories according to their principles, and their applications based on SI are analyzed. Finally, we summarize the application status of SI in the wireless network and discuss the potential prospects of SI algorithms in the application of wireless networks. The wide applicability of SI techniques has exhibited promising prospects in the WSN. Furthermore, the combination of UAV with 5G, mobile edge computing, the IoT, and other technologies presents a direction worthy of development.

## Figures and Tables

**Table 1:** Noun abbreviation.

Abbreviations	Instructions	Abbreviations	Instructions
WSN	Wireless sensor network	UAV	Unmanned aerial vehicle
PSO	Particle swarm optimization	CH	Cluster head
ABC	Artificial bee colony	MS	Master station
BOA	Butterfly optimization algorithm	LP	Label propagation algorithm
FCM	Fuzzy c-means algorithm	BS	Base station
LS	Local search	DBS	Drone base station
GA	Genetic algorithm	VANET	Vehicular ad-hoc network
IoT	Internet of Things	ACO	Ant colony algorithm
GWO	Grey wolf optimization	DBS	UAV as a base station
LEACH	Low energy adaptive Clustering hierarchy	BFO	Bacterial foraging algorithm

**Table 2:** Parameters of ACO algorithm.

Parameter	Meaning
β	relative importance of the heuristic factor
$J_{k}\left(i\right)$	collection of cities that ant k allows selecting in the next step
t	times
$\tau_{ij}\left(t\right)$	pheromone on the edge (i, j)
$\eta_{ij}$	visibility of pheromones on edge (i,j)
$d_{ij}$	distance between cities (i, j)
m	number of ants
k	ant number
n	number of cities
$\Delta \tau_{ij}$	pheromone increment on edge (i, j) of this iteration
$\Delta \tau_{ij}^{k}$	pheromone quantity on edge (i, j) left by the ant k in this iteration
ρ	pheromone evaporation (volatilization) coefficient 1- ρ is the persistence (residual) coefficient where 0 < ρ < 1
$P_{ij}^{k}\left(t\right)$	probability of ant k moving from city i to city j at time t (transition probability)
${\mathrm{tabu}}_{k}$	tabu list for ant k

**Table 3:** CH selection algorithms based on SI.

Method	Year	Key Technology	Uniform (U)/ Random (R)	Static(S)/ Dynamic (D)	Number of Clusters	Size of the Cluster	Multi-hop(M)/Single-hop(S)	CH Selection Factor
LEACH	2000	—	R	S	V	V	S	Cyclic random
[19]	2018	PSO Tabu	R	S	V	V	M	E
[20]	2016	PSO	R	S	F	F	S	E,C
[21]	2016	PSO	R	D	F	V	S	E,D2S,C average intra-cluster distance
[22]	2016	PSO FCM	R	S	F	F	S	-
[23]	2017	PSO BFO	R	S	V	V	S	E
[24]	2019	PSO	R	S	F	V	M	E
[25]	2017	ACO	U	S	F	F	M	Initial: E Below threshold: E,C
[26]	2016	ACO	R	D	F	F	M	E,D2N, LQI
[27]	2017	EACO FABC	U	D	F	F	M	E,C,delay
[28]	2017	ABC FCM	R	D	F	F	S	E,D2S,C
[29]	2017	ABC	R	D	F	V	S	E,D2B

**Table 4:** Routing algorithms of WSN routing protocol based on SI.

Method	Year	Key Technology	Distributed (D)/ Centralized (C)	BestEffort (B)/QoS (Q)	Single Path (S)/Multipath (M) Routing	Event-Driven (E)/Query-Based (Q)	Fault Tolerant	Energy Aware	Loop Free
[19]	2018	PSO Tabu	D	B	S	E	N	N	N
[20]	2016	PSO	D	B	S	Q	N	Y	N
[24]	2019	PSO	D	B	S	Q	N	Y	N
[30]	2016	PSO	D	B	S	QE	N	Y	N
[31]	2017	PSO	D	Q	M	E	Y	N	N
[25]	2017	ACO	D	B	S	E	Y	Y	N
[26]	2016	ACO	D	B	S	QE	N	Y	N
[27]	2017	EACO FABC	D	B	M	E	Y	Y	N
[32]	2017	ACO	D	B	S	E	N	Y	N
[33]	2019	ACO	D	Q	S	E	Y	Y	N
[35]	2017	ACO LP	D	B	S/M	E	N	Y	N
[28]	2017	ABC FCM	C	B	S	Q	N	N	N
[29]	2017	ABC	D	Q	S	E	N	Y	N

**Table 5:** Parameters to be considered in the validation of SI applications in WSNs.

Method	Package Loss	Delay	Packets Received	Run Time	Energy Consumption	Life Time	Active Node Ratio	Other
[19]	Y	Y	N	N	N	N	Y	-
[20]	N	Y	N	N	Y	Y	N	-
[21]	N	N	Y	N	Y	Y	Y	-
[22]	N	N	N	N	Y	N	N	Connection rate
[23]	N	N	N	N	Y	N	N	-
[24]	N	N	N	N	Y	Y	N	Number of hops
[25]	N	N	Y	N	Y	Y	N	-
[26]	N	Y	Y	N	Y	Y	Y	Throughput
[27]	N	N	N	N	Y	N	N	-
[28]	N	N	Y	Y	N	Y	N	Throughput
[29]	N	Y	Y	N	Y	Y	N	-
[30]	N	N	N	N	Y	Y	Y	Throughput
[31]	N	Y	N	N	Y	N	N	-
[32]	N	N	N	N	Y	Y	N	Path length, Packet deliver
[33]	Y	N	N	N	Y	N	N	Routing load, Throughput
[35]	N	Y	N	N	Y	N	N	-
[36]	N	N	N	N	Y	N	N	Coverage rate, Sensing radius of nodes
[38]	N	N	N	N	N	N	N	Deployment cost
[40]	N	N	N	N	N	N	N	Target nodes, Convergence behavior, Location
[41]	N	N	N	N	N	N	N	Node localization

**Table 6:** Simulation platforms analysis.

Simulation Platform	Percentage
Did not mention	32.4
MATLAB	38.3
Java	5.9
Ns2	5.9
OMNeT++/Castalia	8.8
jMetal	2.9
CloudSim package	2.9
QT Creator 2.4.0	2.9

**Table 7:** Experimental simulation scene selections analysis.

Number of Scenarios	Percentage
One or two	61.8
Three or four	17.6
At least five	20.6
